# Modeling of a Generic Edge Computing Application Design

**DOI:** 10.3390/s21217276

**Published:** 2021-11-01

**Authors:** Pedro Juan Roig, Salvador Alcaraz, Katja Gilly, Cristina Bernad, Carlos Juiz

**Affiliations:** 1 Computer Engineering Department, Miguel Hernández University, 03202 Elche, Spain; salcaraz@umh.es (S.A.); cbernad@umh.es (C.B.); 2 Mathematics and Computer Science Department, University of the Balearic Islands, 07022 Palma de Mallorca, Spain; cjuiz@uib.es

**Keywords:** edge computing, fog computing, CNN, formal modeling, ACP, Promela, Spin

## Abstract

Edge computing applications leverage advances in edge computing along with the latest trends of convolutional neural networks in order to achieve ultra-low latency, high-speed processing, low-power consumptions scenarios, which are necessary for deploying real-time Internet of Things deployments efficiently. As the importance of such scenarios is growing by the day, we propose to undertake two different kind of models, such as an algebraic models, with a process algebra called ACP and a coding model with a modeling language called Promela. Both approaches have been used to build models considering an edge infrastructure with a cloud backup, which has been further extended with the addition of extra fog nodes, and after having applied the proper verification techniques, they have all been duly verified. Specifically, a generic edge computing design has been specified in an algebraic manner with ACP, being followed by its corresponding algebraic verification, whereas it has also been specified by means of Promela code, which has been verified by means of the model checker Spin.

## 1. Introduction

Edge computing is a new paradigm that moves computing and storage power from the cloud to the edge of the network, bringing it closer to end devices [1]. This way, the distance between clients and servers gets minimized, achieving better performance regarding latency and jitter, which allows the deployment of highly effective artificial intelligence (AI) processing at the edge of the network [2].

With the exponential growth of the Internet of Things (IoT) devices in recent years, the generation of large-scale data induces issues when forwarding them on to the cloud to be analyzed, which are related to bandwidth overload due to the use of Wide Area Network (WAN) connections, and slow processing times, thus deterring real-time applications, as well as poor security and privacy, because data must travel through WAN links [3].

In order to cope with this, edge computing deals with those concerns by providing high bandwidth, due to the use of Local Area Network (LAN) connections much faster than WAN ones, and extremely-low response times, thus allowing real-time access, along with enhanced security and privacy, as data remains within a campus LAN [4].

**Figure 1:**
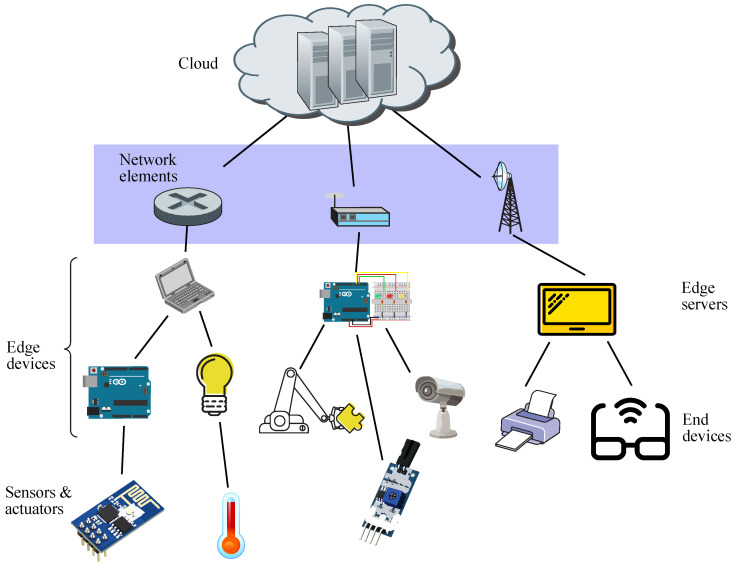
Schematic diagram for edge computing.

Furthermore, edge computing takes advantage of the addition of machine learning (ML) algorithms associated with AI, leading to the concept of Edge AI, which may be seen as the application of AI to run ML tasks on edge devices in order to enhance performance [5]. It is to be noted that an edge device may be either an edge server or an end device, whereas sensors just gather data and forward them on to end devices, whilst network elements connect edge devices to cloud facilities, as shown in Figure 1.

It is worth remarking that edge devices are located in the customer premises and are dedicated to undertaking analysis of a huge amount of data collected by sensors and IoT devices in real-time, whereas cloud data centers may act as backup solutions.

Therefore, the big data generated by the ever-growing amount of IoT devices and mobile computing users will be dealt with on-edge devices powered with AI and using cloud facilities as backup services [6]. In other words, edge computing may be seen as a distributed AI architecture, which mainly makes use of short-distance communications between clients and edge servers, as opposed to long-distance communications to reach the cloud servers, hence reducing bandwidth needs, along with latency and jitter [7].

The goals in this paper are to first review the main applications of edge computing and then to obtain the verification of a generic edge computing design. In fact, different architectures have been proposed for IoT environments [8], although the approach taken herein is a generic high-level one, thus trying to focus on the fundamental building blocks common in most IoT designs and leaving aside the specific implementation details of a particular deployment aimed at a given IoT scenario.

The modeling of that generic edge computing design is to be undertaken by means of two different Formal Description Techniques (FDT), such as a process algebra—called Algebra of Communicating Processes (ACP) [9]—and the modeling language Promela, along with its analysis tool Spin [10]. In each of both cases, a model is presented first, and in turn, an appropriate verification technique is executed.

The purpose of using FDT is to obtain a unified representation method mainly focused on distributed designs run in a concurrent way to check their correctness and improve security. This is carried out by spotting deadlock conditions, such as mutual exclusion, hold and wait condition, no preemption or circular wait, as well as other troublesome conditions like livelock, resource starvation, data race, and priority inversion [11]. It is to be noted that FDT are basically aimed at software developments [12], cyber-physical systems [13], and communication protocols [14].

There are many types of FDT, each one targeting a particular ensemble of concepts within the system being modeled [15]; although, they may all be divided into two broad categories, such as timeless and timed, where the former does not take time into account, hence focusing on qualitative features, whilst the latter does, thus setting the focus on quantitative features [16].

On the one hand, some of the most commonly used techniques within the first category are timeless Petri Nets and timeless process algebras, where ACP is included [17]. In this case, performance may not clearly be measured in time units, which leads to the search for alternative units, as it may be the case of distance specified in the number of links traversed between two given entities.

On the other hand, some of the most usually employed techniques within the second category are queueing networks, timed Petri Nets, and timed process algebras [18]. Furthermore, time-based software simulations may also be included, which is the case of Spin/Promela [19]. In this case, performance may be obviously measured by means of time units.

Therefore, the FDT selected to undertake the models proposed in this paper cover both paradigms, as ACP is a timeless technique, thus carrying out a qualitative study of the scenarios presented while leaving out time constraints. However, Spin/Promela is a timed technique, thus conducting a quantitative study of such scenarios, including time considerations.

Taking all that into account, the contributions of this work are the following:

- Outline on convolutional neural networks;
- Overview of fog computing;
- Overview of edge computing;
- Modeling of generic edge computing in ACP;
- Modeling of generic edge computing in Promela.

The rest of the paper is organized as follows: Section 2 introduces convolutional neural networks, then, Section 3 reviews fog computing environments for IoT devices, after that, Section 4 presents edge computing environments for IoT devices, along with some of its main industry fields of application, next, Section 5 focuses on the verification of a generic edge model with ACP, afterwards, Section 6 carries out the verification of such a model with Spin/Promela, and eventually, Section 7 draws some final conclusions.

## 2. Convolutional Neural Networks

Regarding AI, it may be considered as machine intelligence, as opposed to human intelligence [20]. Specifically, ML may be seen as a subset of AI where a machine has the ability to learn to undertake tasks as well as to keep improving its performance without human intervention [21]. ML functionality is two-fold, such as training for a task, and in turn, running that task, where the former is defined by the quick application of knowledge and training through huge data sets, whilst the latter is done by executing pattern recognition and predicting future patterns.

In this sense, deep learning (DL) may be deemed as a subset of ML where the techniques being used are organized into neural networks so as to simulate the process of decision-making in humans, hence requiring a massive number of parameters [22]. Besides, DL has a layered structure, where each layer accumulates the knowledge of the previous. If ML and DL are to be compared, the latter requires a larger dataset and more intensive computation when training a model, whilst getting much better accuracy when predicting the outcome and learning new features in an automatic way.

It is to be noted that DL is the foundation of artificial neural networks (ANN), which take the notion of neurons from the human brain (with its axons and dendrites), thus working by interconnecting and replicating signals like real neurons [23]. Moreover, all connections have a weight associated with their importance, which allows the networks to learn as values change. Focusing on a single neuron, its corresponding input values are a weighted sum, and the result is passed on to an activation function, whose outcome will dictate whether each input value is forwarded on.

ANN are formed of three layers, input, hidden, and output; where the first one brings raw data in, the middle one processes it, and the last one delivers the outcome [24]. ANN may be further classified as shallow if there is only one hidden layer, or deep if there are more than one, which are also referred to as deep neural networks (DNN).

This three-layer distribution leads to ANN also being known as feed-forward neural networks because of the direction of processing, which provokes challenges in some scenarios, such as capturing sequential information or solving image classification [25]. The former is fixed by recurrent neural networks (RNN), even though spatial relationships are better handled by convolutional neural networks (CNN). However, they all suffer some degree of the vanishing and exploding gradient problem during the backpropagation [26], leading to slow learning and unstable learning, respectively.

As a summary, it may be said that ANN are better fitted for text or tabular data, whilst RNN are for audio and sequence data, and CNN are for image and video data. Furthermore, CNN outperforms the rest, which is why it is used most commonly, but at the expense of using larger data sets and training times [27]. On the other hand, ANN is a better tool when data are limited, and RNNs like long short-term memory (LSTM) and GRU (gated recurrent units) are quite popular in natural language models and text-to-speech tasks; whereas CNN fits all contexts at the expense of higher computing resources and longer training times [28].

Focusing on CNN, they are based on filters, also known as kernels, aimed at extracting the relevant features out of the input data by means of the convolution operation. Basically, convolving an input with filters extracts feature maps. The main advantages of using CNN are automatically learning the filters to be used and precisely capturing spatial features thanks to the concept of parameter sharing [29]. However, CNN presents some disadvantages when data differ from that in the dataset regarding rotation, reflection, translation, or rescaling [30], hence requiring further processing to sort it out. Therefore, some specific features may be added up to CNN in order to achieve the appropriate customizable accuracy degree, depending on whether the target is as complex as building up a polarimetric synthetic aperture radar (PolSAR) [31] or otherwise playing rock-paper-scissors [32].

Visual recognition tasks are well suited applications of CNN, with image classification being considered one of the most prominent, leading to object detection, localization, or semantic segmentation [33]. Much research has been undertaken in this field so as to achieve better results, containing different combinations of convolution layers, pooling layers, fully connected layers, batch normalization units, or rectified linear units to minimize the error rates in image recognition [34]. Some of the most relevant milestones in CNN for image recognition are LeNet-5 in 1989, AlexNet in 2012, ZFNet in 2013, VGG in 2014, GoogLeNet in 2014, ResNet in 2015, and DenseNet in 2016 [35].

In this sense, some enhancements of CNN have been proposed for specific duties in recent times, to obtain greater accuracy in predicting visual recognition in data science, such as subpixel displacement measures [36], defect identification in high-speed trains [37], correlating image-like data out of quantum systems [38], modeling wind field downscaling [39], designing a zero knowledge proof scheme [40], classifying satellite image time series [41], working with ensembles [42], dealing with osteoporosis diagnoses [43], screening and staging diabetic retinopathy [44], analyzing cloud particles [45], inspecting diffraction data [46], or examining x-ray images [47].

## 3. Fog Computing and IoT

An interesting attempt to cope with the outburst of an ever increasing number of IoT devices is fog computing, which proposes a hierarchical distributed architecture, extending from the edge to the cloud [48]. This way, the remote processing and storage power located on the cloud facilities in the cloud paradigm are drawn near the end user, somewhere between the edge and the cloud, inducing a reduction of the levels of bandwidth and latency when undertaking remote computing tasks and having the cloud as a backup, thus achieving better performance than working solely with the cloud [49].

**Figure 2:**
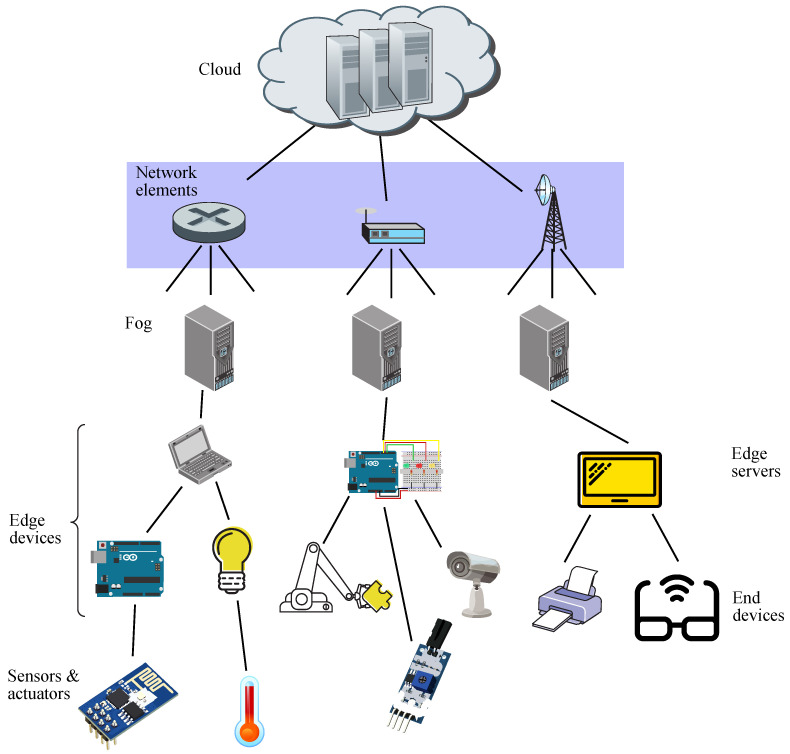
Schematic diagram for fog computing.

There is conceptual difference in paradigms between edge and fog, such as the former performs computing at the edge of the network (being in the end device itself or in the edge server), whereas the latter does it anywhere between the edge of the network and the cloud (even though the typical location is the edge itself) [50]. This fact may be appreciated in Figure 2, where fog facilities are located somewhere before the cloud [51].

Hence, a fog infrastructure may be considered as an integration of some capabilities coming down from the cloud and other capabilities coming up from IoT devices, thus getting better performance and efficiency [52]. In this context, the whole fog ecosystem may be seen as composed by three strata, such as edge devices, fog, and cloud, allowing the components of each fog application to be deployed in the most convenient stratum, related to the type of task run by each component or the latency rate to be expected [53].

On the other hand, the distribution of IoT devices in both the spatial and temporal domains are not usually uniform, especially in the moving IoT environments [54]. This circumstance usually leads to unbalanced loads of traffic, which may be alleviated by the implementation of offloading policies among fog nodes [55], or any other feasible resourceful environment, as a cloud node [56]. Furthermore, the issues of provisioning resources for IoT devices and dynamically adapting to the requirements of IoT applications and services may be addressed by implementing an orchestrator [57].

Moreover, security and privacy issues may arise when deploying a fog environment, which may be dealt with in different ways, such as implementing encrypted communications [58], or using blockchain technology [59]. The issues may be classified into three broad categories, such as network services and communications to interconnect IoT devices with fog nodes, data processing to manage the big data analytics and distribution in fog nodes, and IoT device privacy to apply secure policies related to data, identities, locations, and usage [60].

One of the main challenges in fog computing is energy consumption, as the great amount of IoT devices may be involved in a huge quantity of data transfers with the fog nodes, each of those involving its own particular level of quality of service [61]. In order to cope with that, the search for energy-aware strategies to minimize its consumption rate is being encouraged, such as applying a dynamic energy control [62].

It is to be noted that the integration of fog computing with AI techniques, namely fog AI, makes it possible to bring intensive calculation tasks near the end user, thus allowing near-real-time applications [63]. In this sense, fog and edge applications oftentimes overlap, as both implement AI methods, which enhance processing capabilities, while providing lower latency rates and allowing higher bandwidth usage than their cloud counterparts [64].

Fog deployments have been implemented in many fields where processing a vast amount of data with strict time constraints renders cloud computing unfit [65]. One scenario may be the analysis of physiological data from wearable devices (the concept of smart health), where such devices obtain relevant health-related data from a patient, and in turn, pass them on to fog servers to undertake the processing and respond within a restricted time interval, whilst having cloud servers for backup purposes [66].

Furthermore, data coming from moving vehicles may also benefit from services hosted on fog infrastructures [67] to facilitate driving by scanning changing traffic conditions and suggesting the most adequate path, while ensuring safety by checking sensors measuring the vehicle variables; these conditions may lead to autonomous driving [68].

Another interesting use may be the application of the IoT paradigm to improve industrial tasks, leading to the concept of industry 4.0 [69]. This way, industrial manufacturing becomes both intelligent and efficient by providing industrial fieldbuses to machines, appliances, and robots. At the same time, it assures a secure environment to protect sensitive data generated during the industrial operability [70].

On the other hand, the concept of trust may help identify and isolate rogue fog nodes, as those may collect data in an unauthorized manner or manipulate data in transit, thus committing security and privacy breaches [71]. To cope with that, cryptographic solutions may ease those issues if external attacks are perpetrated, coming from unauthenticated fog nodes. However, they are useless regarding internal attacks, hence, coming from fog nodes already authenticated operating within the network [72]. Therefore, fog nodes are able to communicate with other nodes where a fog level trust has been properly established [73].

Trust evaluation may be imposed by different means, such as reputation-based, plausibility-based, trusted isolated environment, secure element, or trusted platform module [74]. On the other hand, some well-known trust-related issues are denial of service, man in the middle, and collusion attacks, which may be avoided by using the proper countermeasures based on trust [75].

Trust indicators may contain two notions, the subjective one (focused on the individual interests of user’s interactions), and the objective one (depending on individual interaction experiences) [76]. The former is obtained from the feedback obtained out of multiple sources; whereas, the latter is done based on the quality of services, where an overall trust value determines the trustworthiness of each node, resulting in the establishment of a trusted environment [77].

In this sense, trust management may involve both, proving that a given entity is trustworthy to others, while the others are trustworthy to a given entity. The establishment of a trust relationship may significantly reduce risks, even though it may be considered context-dependent, as trust rates might vary in diverse situations [78]. Furthermore, blockchain technology is a suitable tool to build up distributed trust architectures [79].

## 4. Edge Computing and IoT

An alternative way to deal with the ever-increasing amount of IoT devices is edge computing, which is a distributed architecture where computation occurs close to the network edge. It is also known as multi-access edge computing (MEC) as a result of bringing together the cloud computing capabilities at the edge of the network with a radio access network (RAN) [80]. The latter handles the connections of IoT devices through any type of wired or wireless connection, and the former is in charge of managing the massive volume of heterogeneous data generated by those IoT items [81].

The main characteristics of MEC environments are similar to those of fog ecosystems, such as close geographical distribution, mobility support, location awareness, heterogeneity, offloading, ultra-low latency, and high bandwidth [82]. Therefore, it results in advantages regarding performance, efficiency, reliability, privacy, and security.

However, it seems that lately there is a prevalence in developments related to edge computing as opposed to fog computing ones, as is the case of Industrial Internet of Things [83]. In this field, cyber-physical systems (CPS) and digital twins (DT) have been popularized. The former being systems made of a combination of physical and digital components working hand in hand, whilst the latter being a virtual representation acting as the digital counterpart of a physical entity [84].

Regarding the scope, cloud computing works in the digital domain, being primarily focused on data centres, whereas edge computing covers the cyber-physical domain and also touches its digital counterpart because of the data centres, whilst fog computing is located in the middle of both domains [85]. On the other hand, neither work in the physical domain, as they are all related to computing, although edge is the closest.

MEC is standarized by ETSI GS MEC 003 V2.2.1 (2020-12), stating that MEC applications are software-only entities running on top of a virtualization infrastructure [86]. Moreover, it defines a MEC framework where three different levels may be defined, such as MEC system level on top (including user applications and mobile edge orchestrator), MEC host level in the middle (containing MEC platform, MEC applications and the virtualization infrastructure, all three composing the MEC host, apart from the host level management), and the network level (embracing the network connections involved) [87].

Furthermore, MEC may be seen under three different functional views, such as infrastructural, applicational, and operational [88]. The first one is composed by a computing node working as the host and a guest system usually made of containerized software. Meanwhile, the second one is comprised by the components needed to meet the application requirements; whereas, the third one is devoted to managing the edge node during its lifecycle stages, going from the planning all the way to the retirement [89].

Additionally, the demand for MEC services is supposed to be driven by both the introduction of 5G cellular telephony and the need for distributed data processing power [90], forecasting a huge growth in the coming years. However, some challenges are faced ahead, such as a common naming scheme for IoT devices, as well as a standarize way to undertake programmability and managament due to the IoT’s heterogeneous nature [91].

### 4.1. Edge AI

With respect to the edge computing scenarios, they may be classified into three broad categories. First, latency-sensitive applications, such as VR/AR/MR games, self-driving cars, and industrial IoT. Second, data-intensive applications, such as processing video for IoT systems or dealing with high volumes of sensing data collected by IoT devices. Third, privacy-sensitive applications, such as traffic related to protected health registers, personally identifiable data, or any type of personal sensitive information [92].

Anyway, edge computing scenarios with the integration of AI, namely edge AI, drive the rising of different types of MEC applications where the AI is located at the edge of the network, as opposed to AI in the cloud, namely cloud AI. This way, edge AI provides a fast response and autonomy for the local environments of IoT deployments whilst cloud AI facilitates a thorough analysis focused on the whole IoT ecosystem [93].

The popularization of edge AI along with the integration of cloud AI as a backup and storage service make it possible to carry out the processing of massive amounts of sensing data coming from IoT devices in all kinds of environments. This leads to the convergence of AI and IoT, also known as AIoT [94]; thus, enhancing the computational tools for dealing with big data derived from IoT-based devices in basically any field [95].

Moreover, edge intelligence, which is another name for edge AI, might be further divided into AI for edge and AI on edge. The former focuses on providing AI technologies aimed to boost edge computing capabilities, whilst the latter studies how to better apply model training and inference to construct AI models on the edge [96].

Hence, deploying AI-powered applications on the edge may raise the effectiveness of MEC applications compared to their cloud counterparts regarding real-time analytics and monitoring [97], as well as smart manufacturing, process automation, and data storage [98]. As a consequence, IoT devices may take full advantage of MEC applications with a cloud backup, thus providing IoT sensors and actuators with applications and services in several vertical domains, being customizable according to specific requirements [99].

Among those possible services with edge technologies, one of the most popular and relevant uses is AI-based real-time video analytics. In this context, many solutions are available, such as the one being deployed by Singapore’s government in order to tackle the spread of the covid-19 pandemic [100], aimed at targeting face mask detection, social distance analyzer, crowd density control, and even person searching and retrieval.

### 4.2. Edge Computing Applications

MEC and AI establish a mutually beneficial relationship in many aspects, such as increasing performance related to resource management or scheduling [101]. However, MEC applications powered by AI achieve huge advances in different domains related to IoT, such as smart multimedia, smart transportation, smart city, or smart industry [102].

Regarding smart wearable devices, their popularity has rapidly increased lately due to their wearability [103]. Their light weight and compact size limit their computing capabilities; thus, MEC applications may offer a great range of possibilities to increase their computing power [104]. Moreover, those wireless sensing devices have been shown to operate properly even in harsh conditions [105]. They are widely used in health care, leading to defining Internet of Medical Things (IoMT) [106], as well as other tasks related to tracking activities such as sports, rehabilitation, or human-robot collaboration [107].

With respect to smart health, it focuses on classifying health data related to vital sign monitoring and fall detection [108]. In this sense, there are many different types of wireless medical body sensors to obtain vital patient data, such as pressure or implantable sensors [109]. However, other sorts of sensors are equally important, such as those used in operation rooms, emergency rooms, or intensive care units [110], or otherwise, in ambient assisted living scenarios [111]. Furthermore, the interaction with cloud facilities for analytics and storage improves the overall performance, as it greatly reduces the costs of treatment and enables a personalized medical service at any time and place [112].

With regard to industry 4.0, also known as industrial internet of things (IIoT), it interconnects numerous variable industrial equipment devices through the network [113]. This way, data acquisition and exchange, as well as collaborative decision-making, are carried out through distributed computation in near-real-time [114]. Hence, efficient, intelligent, and decentralized solutions are available [115], allowing the interaction of multivendor devices through heterogeneous networks in an optimal manner [116], where a higher level of trust may be imposed by using blockchain technology [117]. It is to be remarked that Industry 4.0 is considered to be a new paradigm called the fourth industrial revolution, which the world is just coming into. The first industrial revolution was related to mechanization, the second one to mass production and electricity, the third one to automation and computing, and the forth one to cyber physical systems and IoT [118].

However, the fifth industrial revolution, known as industry 5.0, is already emerging and is focused on the personalized demand of customers [119]. Basically, it applies AI solutions to extreme automation and hyperconnectivity in order to democratize knowledge coproduction [120]. In this sense, AIoT technology is a key player as it provides an optimal immersive experience in real-time interactions, no matter whether they are machine-machine, human-machine or human-human [121]. However, the use of edge AI may lead to the consumption of additional energy, even though in order to reduce the carbon footprint, the concept of green IoT (G-IoT) has been introduced to lower the greenhouse effect provoked by the Edge-AI G-IoT systems. Thus, leveraging the adoption of the digital circular economy (DCE) concepts to achieve a sustainable development regarding economic, social, well-being, and environmental dimensions [122]. Therefore, this new paradigm will be the relevant driving force to achieve a smart, green, sustainable, resilient, and human-centric world [123].

Another important field is vehicular edge computing (VEC), which incorporates edge AI to increase the computing capacity of vehicular ad hoc networks (VANET) [124]. This way, AI-powered services are hosted close to smart vehicles; hence, improving quality of service (QoS) and reducing latency [125]. VANETs are composed by two basic elements, such as smart vehicles and roadside units aimed at facilitating network access and providing services, such as road safety, traffic efficiency, or added-value applications, such as infotainment or interactive tasks [126]. Some relevant challenges in high mobility, time-sensitive, and computation-intensive scenarios are related to security and privacy in both vehicle-to-vehicle (V2V) and vehicle-to-roadside units (V2R) [127]. Challenges are also related to the cost-efficient task of offloading as resources are likely to be transferred among edge and cloud domains due to the traffic conditions at a given place and time [128].

It is to be noted that models employed on edge AI facilities must first be properly trained. Such models have been typically trained under the orchestration of a central server, known as parameter server (PS), where edge devices forward all raw training data (local datasets) to be aggregated there for training [129]. This paradigm is known as centralized learning (CL) and may cause issues related to data protection regulations [130]. However, a new paradigm called federated learning (FL) may preserve privacy issues by keeping the raw training data decentralized on the edge devices and forwarding just the locally computed model parameters to the PS, which in turn performs the model aggregation with all stuff received, and then updates the model to the edge devices [131]. A typical instance of FL is represented by a scenario where all edge devices calculate gradients on their own local dataset and forward them on to the PS, which in turn processes all those gradients and forwards the updated weights back to the edge devices for them to update the model [132]. Furthermore, FL proves to be more effective in communication overhead with a small performance loss in learning accuracy, even though a scenario with hybrid federated centralized learning (HFCL) may partly compensate for such a loss [133], where the PS sends the same updates to all.

Likewise, serverless edge computing as an application development and deployment model for IoT devices is on the rise. Here, the developer just supplies the core function code (function as a service, or FaaS) whilst the behind-the-scenes aspects are delivered by the provider (backend as a service, or BaaS) [134]. Although the serverless paradigm was designed for cloud environments, it has been adapted to edge domains in order to incorporate its advantages, such as deleting always-on services, which provoke high electricity usage, even though its cloud-driven design may pose drawbacks [135]. On the other hand, the serverless edge-based IoT deployments integrated with the cloud for offloading purposes may succeed in reducing overall execution times and obtaining classification accuracy [136]. Additionally, if a warm-start deployment mode is used, then the FaaS platform always has available resources; whereas, a cold-start deployment mode’s modules are deleted after its execution, thus bringing resource and cost savings [137]. Furthermore, latency-aware IoT applications may also take advantage of this paradigm by applying queueing prioritazion, dynamic workload allocation, and resource reclamation methods to reassign them from the over-provisioned functions to under-provisioned ones [138].

## 5. ACP Model

There are many different deployments of edge computing, depending on the different types of sensors and actuators being used, each one with its own characteristics and specifications, as well as the interconnections of the servers being employed, leading to diverse network topologies. However, putting the focus on a generic high-level representation of an edge computing implementation, it is possible to achieve an abstract framework where a block diagram may be designed with concrete examples that fit into it.

Traditional communication architectures, such as client-server or peer-to-peer, do not fit properly into IoT deployments, as those devices have constraint resources regarding computing and power. Otherwise, publisher/subscriber paradigm (Pub/Sub) better meet IoT requirements, as there is central a server, known as a broker, dealing with a group of end devices connected either through wired or wireless links [139].

In Figure 1, brokers are represented by edge servers located at the edge of the network, whereas publishers are end devices connected to sensors, whilst subscribers are also end devices, even though they are connected to actuators. This way, sensors read data from the external environment (such as measuring temperature or humidity) and pass those raw data on to the system under a given topic through an end device acting as a publisher. When those raw data reach the edge server, the broker tries to process them, and if it succeeds, then it forwards them on to the end devices acting as subscribers associated to that topic, which in turn, send those processed data to actuators, which execute the commanded actions on the external environment (such as setting an HVAC mechanism or an alarm). Otherwise, if the broker does not succeed, then it passes the data to a higher processing level, such as the cloud [140].

The aforementioned figure shows a network connection layer which only routes traffic flows from the edge servers (brokers) up to the cloud and the other way around, thus not taking part in remote computing. Hence, the processing entity above an edge server is the cloud, which in fact, acts as the only hierarchical entity for edge servers when dealing with offloading or backup processing and storage. Otherwise, in a fog environment, the fog nodes may be located between the edge of the network and the cloud, and in such a case, edge servers will be connected to fog servers, those being the next and higher processing level, which in turn, will be connected to cloud servers, these being the last and highest processing level, staying on top of the hierarchy [141].

The features described above may be represented by means of modeling the behavior of each component using a range of FDT, each one focusing on different characteristics. In this sense, a good candidate may be ACP, which is an abstract untimed process algebra aimed at reasoning about relationships among process terms, leaving apart their real nature [142]. ACP modeling starts with the specification of the entities composing a concurrent model so as to obtain its ACP specification when applying the proper operators. This may be further verified if the algebraic expressions for the behavior of the real system and that of the model contain the same string of actions and the same branching structure, thus being referred to as rooted branching bisimilar [143].

In order to undertake ACP modeling for communicating processes, two atomic actions are needed, such as sending a message *d* to a channel *i*, denoted by $s_{i}\left(d\right)$, and receiving a message *d* from a channel *i*, stated by $r_{i}\left(d\right)$. Moreover, there are some operators to deal with those atomic actions, such as the sequential one, given by the · sign, the alternate one, exposed by the + sign, the concurrent one, depicted by the || sign, and the conditional one, exhibited by the expression (true◃condition▹false). Additionally, two extra operators are usually employed when it comes to work out specifications and verifications, such as the encapsulation one, named by $\partial_{H}$, so as to promote internal communications ($c_{i}$) whilst cancelling internal atomic actions ($s_{i}$ and $r_{i}$), and the abstraction one, named by $\tau_{I}$, so as to mask internal actions and communications, thus prevailing the external actions, which unveils the external behavior of the model.

Taking this all into account, two scenarios are modeled, where the first one is related to an edge environment and the second one is associated with a fog environment. Both have sensors and actuators external to the model, and a channel directly connected to it, specifically to the end devices, which in turn, have channels to interconnect them to the edge servers. However, in the edge domain, the edge servers connect straight to the cloud servers, whereas in the fog domain, the edge servers link to the fog servers, and those do to the cloud servers.

Furthermore, it is going to be supposed that edge facilities are integrated with AI, namely Edge AI, even though CNN are supposed to be already trained, hence CNN are going to be executed when needed. However, CNN are internal functions in all types of servers, being represented by different greek letters depending on the location of the server and accepting as many parameters as the channels coming in. Furthermore, CNN will not be eventually taken into account by ACP models because they will be masked when applying the abstraction operator for being internal functions.

Therefore, two different type of models are studied herein, using ACP. Two case scenarios are shown—an edge computing one, where three levels are taken into consideration (end devices, edge servers, and cloud); and a fog computing one, where a fog level is summed up. Regarding ACP [144], the models proposed will be exhibited by means of algebraic expressions to portray the behavior of the concurrent communicating processes involved, containing the specifications and verifications, whilst respecting Spin [145], the models presented will be exposed by means of Promela code [146], including the verification by means of the Spin model checker, along with some message sequence charts (MSCs) describing the message exchanges performed by communicating concurrent processes involved in a visual way.

### 5.1. Edge Scenario

**Figure 3:**
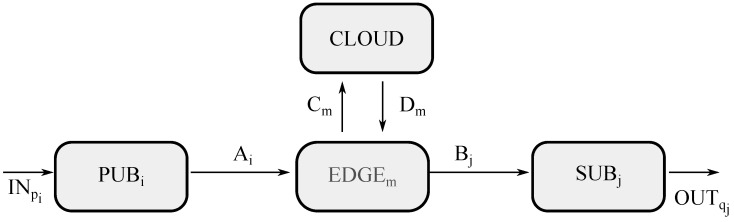
Model for edge computing in ACP.

This first scenario is exhibited in Figure 3, where four different type of entities may be appreciated, such as a group of publishers (represented by $PUB_{i}$), a group of edge servers (represented by $EDGE_{m}$), a group of subscribers (represented by $SUB_{j}$), and cloud premises (represented by CLOUD).

To start with, the channel getting into the model is called $IN_{p_{i}}$, meaning the channel through which a sensor forwards raw data ($d_{p_{i}}$) on to the system, where *p* is related to the sensor identifier and *i* is referred to as the sending end device getting the raw data (*d*) into the system, which is also known as publisher *i* or $PUB_{i}$ in the diagram. After receiving the data from channel $IN_{p_{i}}$, publisher *i* will carry out a unitary processing of the data to encapsulate them according to the communication protocol used, and assign them the corresponding topic by means of function $\phi (d_{p_{i}})$, sending them over to the edge server *m* (also known as $EDGE_{m}$), through channel $A_{i}$.

At this point, edge *m* will undertake an aggregated processing with all data being received by means of the edge CNN $\theta (d_{1_{m}}\cdots d_{{max}_{m}})$. This results in either sending the processed data ($e_{q_{j}}$)—where *q* is related to the actuator identifier, *j* is the receiving-end device getting the processed data (*e*), *j* or $SUB_{j}$ in the diagram is subscriber or receiving-end device with appropriate topic, $B_{j}$ is the channel—or forwarding them up to a cloud, which is also labeled as CLOUD, through channel $C_{m}$.

Then, the servers in the cloud facilities will handle an aggregate processing with all data obtained through all edges by means of the cloud CNN $\psi ({⋃}_{m}\left\{d_{1_{m}}\cdots d_{{max}_{m}}\right\})$ to calculate what to do, resulting in forwarding the processed data to the proper edge *m* (no matter whether it is the same edge server as before or otherwise) through channel $D_{m}$. This, in turn, sends data to the proper subscriber with the adequate topic through channel $B_{j}$ without much processing at the edge as the cloud already did so.

Eventually, when a subscriber *j* receives processed data in channel $B_{j}$, then it carries out a unitary processing of those data to decapsulate them by means of the function $\phi (e_{q_{j}})$, so as to send them to the proper actuator, thus leaving the model through channel $OUT_{q_{j}}$.

As a side note, all entities need to always be ready to go, and for that reason, the algebraic expressions obtained are all guarded linear recursive.

Furthermore, the whole system may be seen as a hub and spoke network topology with different levels of hubs, whose layout might be compared as to how a DNS (domain name system) server makes recursive queries on behalf of clients.

In summary, here they are the ACP model of the entities described, such as $PUB_{i}$ in Equation (1), $EDGE_{m}$ in Equation (2), CLOUD in Equation (3), and $SUB_{j}$ in Equation (4).

(1) $$PUB_{i}=\left(r_{IN_{p_{i}}}\left(d_{p_{i}}\right)\cdot \phi \left(d_{p_{i}}\right)\cdot s_{A_{i}}\left(d_{p_{i}}\right)\right)\cdot PUB_{i}$$

(2) $$\begin{array}{c} EDGE_{m}=(r_{A_{i}}\left(d_{p_{i}}\right)\cdot \left(s_{B_{j}}\left(e_{q_{j}}\right)◃\;\theta \left(d_{1_{m}}\cdots d_{{max}_{m}}\right)\;▹s_{C_{m}}\left(d_{1_{m}}\cdots d_{{max}_{m}}\right)\right)+\\ r_{D_{m}}\left(e_{q_{j}}\right)\cdot s_{B_{j}}\left(e_{q_{j}}\right))\cdot EDGE_{m} \end{array}$$

(3) $$CLOUD=\left(r_{C_{m}}\left(d_{1_{m}}\cdots d_{{max}_{m}}\right)\cdot \psi \left(\underset{m}{⋃}\left\{d_{1_{m}}\cdots d_{{max}_{m}}\right\}\right)\cdot s_{D_{m}}\left(e_{q_{j}}\right)\right)\cdot CLOUD$$

(4) $$SUB_{j}=\left(r_{B_{q_{j}}}\left(e_{q_{j}}\right)\cdot \phi \left(e_{q_{j}}\right)\cdot s_{OUT_{q_{j}}}\left(e_{q_{j}}\right)\right)\cdot SUB_{j}$$

At this point, all the ACP models may be run in a concurrent manner, as they are all non deterministic, and the encapsulation operator may be applied in order to attain the sequence of events in a timely fashion, as in Equation (5). In this sense, it is to be noted that the encapsulation operator reveals the internal communications happening in all internal channels within the model, thus leading their related atomic actions to deadlock. It is to be pointed out that the symbol ∅ in a conditional operator stands for doing nothing in case of that option being selected, meaning that the corresponding CNN has calculated the path to get to the destination, making it unnecessary to send data to the cloud for further calculations.

Basically, Equation (5) exposes what happens with raw data $d_{p_{i}}$ coming from sensor *p* in the external environment through channel $IN_{p_{i}}$, which gets them into the system at Publisher *i*, which then carries out a unitary processing $\phi (d_{p_{i}})$ and forwards them through channel $A_{i}$ towards an Edge *m*, which in turn, aggregates such data with its own parameters $\left(d_{1_{m}}\cdots d_{{max}_{m}}\right)$ and processes them with its edge CNN $\theta (d_{1_{m}}\cdots d_{{max}_{m}})$, containing its edge model setup.

At that stage, if this edge CNN is able to deal with those raw data, it will send the resulting processed data $e_{q_{j}}$ through channel $B_{j}$ towards Subscriber *j*, which undertakes a unitary processing $\phi (e_{q_{j}})$ and sends them out of the system through channel $OUT_{q_{j}}$ towards Actuator *q* in order for it to act on the environment. Otherwise, if this edge CNN is not able to deal with those aggregated data, they are forwarded through channel $C_{m}$ towards the cloud, which compiles all aggregated data coming from all edges $\left({⋃}_{m}\left\{d_{1_{m}}\cdots d_{{max}_{m}}\right\}\right)$ and will use its cloud CNN $\psi ({⋃}_{m}\left\{d_{1_{m}}\cdots d_{{max}_{m}}\right\})$ that contain its cloud model setup to deal with compiled data, which in turn, will send the processed data back through channel $D_{m}$ towards subscriber *j*, which does the same actions as in the other option so as to forward the processed data to the actuator *q*.   

(5) $$\begin{array}{c} \sum_{i}\sum_{j}\sum_{p}\sum_{q}\sum_{m}\partial_{H}\left(PUB_{i}||EDGE_{m}||CLOUD||SUB_{j}\right)=\\ (r_{IN_{p_{i}}}\left(d_{p_{i}}\right)\cdot \phi \left(d_{p_{i}}\right)\cdot c_{A_{i}}\left(d_{p_{i}}\right)\cdot \\ \left(\emptyset ◃\;\theta \left(d_{1_{m}}\cdots d_{{max}_{m}}\right)\;▹\left(c_{C_{m}}\left(d_{1_{m}}\cdots d_{{max}_{m}}\right)\cdot \psi \left(\underset{m}{⋃}\left\{d_{1_{m}}\cdots d_{{max}_{m}}\right\}\right)\cdot c_{D_{m}}\left(e_{q_{j}}\right)\right)\right)\cdot \\ c_{B_{q_{j}}}\left(e_{q_{j}}\right)\cdot \phi \left(e_{q_{j}}\right)\cdot s_{OUT_{q_{j}}}\left(e_{q_{j}}\right))\cdot \partial_{H}\left(PUB_{i}||EDGE_{m}||CLOUD||SUB_{j}\right) \end{array}$$

At that moment, the abstraction operator may be applied to obtain the external behavior of the model, as in Equation (6). It is to be noted that the abstraction operator masks all internal actions as well as all internal communications.

(6) $$\begin{array}{c} \sum_{i}\sum_{j}\sum_{p}\sum_{q}\sum_{m}\tau_{I}\left(\partial_{H}\left(PUB_{i}||EDGE_{m}||CLOUD||SUB_{j}\right)\right)=\\ r_{IN_{p_{i}}}\left(d_{p_{i}}\right)\cdot s_{OUT_{q_{j}}}\left(e_{q_{j}}\right)\cdot \tau_{I}\left(\partial_{H}\left(PUB_{i}||EDGE_{m}||CLOUD||SUB_{j}\right)\right) \end{array}$$

Otherwise, the external behavior of the real system may also be expressed by means of ACP, as in Equation (7), such that some raw data (*d*) from a sensor through an incoming channel ($r_{IN}\left(d\right)$) gets processed anywhere in the system (that being an edge server or a cloud server) to obtain the processed data (*e*), which eventually goes out to an actuator through an outgoing channel ($s_{OUT}\left(e\right)$).

(7) $$X=r_{IN}\left(d\right)\cdot s_{OUT}\left(e\right)\cdot X$$

By comparing expressions Equations (6) and (7), it is clear that both are recursive expressions multiplied by the same factors. Therefore, it may be said that both are rooted branching bisimilar, because they present the same actions and the same branching structure, so Equation (8) applies.

(8) $$\sum_{i}\sum_{j}\sum_{p}\sum_{q}\sum_{m}\tau_{I}\left(\partial_{H}\left(PUB_{i}||EDGE_{m}||CLOUD||SUB_{j}\right)\right)⟷X$$

Hence, this is a sufficient condition to get a model verified, thus the proposed ACP model for edge computing may be considered verified.

### 5.2. Fog Scenario

**Figure 4:**
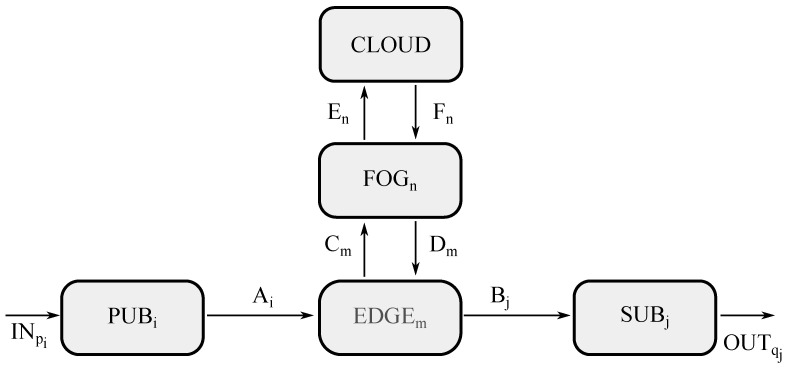
Model for fog computing in ACP.

This second scenario is exhibited in Figure 4, where five different types of entities may be appreciated, such as a group of publishers (represented by $PUB_{i}$), a group of edge servers (represented by $EDGE_{m}$), a group of subscribers (represented by $SUB_{j}$), and cloud premises (represented by CLOUD), as well as a group of fog servers (represented by $FOG_{n}$), which is the difference from the previous scenario.

This schematic diagram is quite similar to that corresponding to the edge computing case, where the only differences are the channels coming into and going off the fog block, as well as the CNN handling the aggregated processing at the fog level, which receives raw data from the edge level, thus being portrayed by $\chi ({⋃}_{m}\left\{d_{1_{m}}\cdots d_{{max}_{m}}\right\})$, whereas the CNN managing the aggregated processing at the cloud level now receives raw data from the fog level, thus being represented as $\psi ({⋁}_{n}\left\{{⋃}_{m}{\left\{d_{1_{m}}\cdots d_{{max}_{m}}\right\}}_{n}\right\})$.

On the contrary, CNN, carrying out the aggregated processing at the edge level remains the same, which receives raw data from the end device level, thus being illustrated by $\theta (d_{1_{m}}\cdots d_{{max}_{m}})$. Meanwhile, unitary processing performed at the end devices stays the same, such as $\phi (d_{p_{i}})$ in the publishers for raw data and $\phi (e_{q_{j}})$ in the subscribers for processed data. Furthermore, external channels and those within the edge level go unchanged.

Here, the ACP model of the entities are described, such as $PUB_{i}$ in Equation (9), $EDGE_{m}$ in Equation (10), FOG in Equation (11), CLOUD in Equation (12), and $SUB_{j}$ in Equation (13). In this framework, it is to be said that Equation (9) is analogous to Equation (1), as well as Equations (10) to Equation (2), and Equations (13) to Equation (4). Furthermore, Equation (11) is similar to Equation (10) as both maintain the same logic about trying to solve an upcoming request, although each one has its own type of data and channels involved. If that is the case, the processed data is forwarded down the hierarchy on the way to reach the proper actuator, whereas on the contrary, the aggregated data is forwarded up the hierarchy towards a more powerful entity to carry out the processing. Additionally, Equation (12) is similar to Equation (3), even though each one has different sorts of data and channels involved.

(9) $$PUB_{i}=\left(r_{IN_{p_{i}}}\left(d_{p_{i}}\right)\cdot \phi \left(d_{p_{i}}\right)\cdot s_{A_{i}}\left(d_{p_{i}}\right)\right)\cdot PUB_{i}$$

(10) $$\begin{array}{c} EDGE_{m}=(r_{A_{i}}\left(d_{p_{i}}\right)\cdot \left(s_{B_{j}}\left(e_{q_{j}}\right)◃\;\theta \left(d_{1_{m}}\cdots d_{{max}_{m}}\right)\;▹s_{C_{m}}\left(d_{1_{m}}\cdots d_{{max}_{m}}\right)\right)+\\ r_{D_{m}}\left(e_{q_{j}}\right)\cdot s_{B_{j}}\left(e_{q_{j}}\right))\cdot EDGE_{m} \end{array}$$

(11) $$\begin{array}{c} FOG=(r_{C_{m}}\left(d_{1_{m}}\cdots d_{{max}_{m}}\right)\cdot \\ \left(s_{D_{m}}\left(e_{q_{j}}\right)◃\;\chi \left(\underset{m}{⋃}\left\{d_{1_{m}}\cdots d_{{max}_{m}}\right\}\right)\;▹s_{E_{n}}\left(\underset{m}{⋃}\left\{d_{1_{m}}\cdots d_{{max}_{m}}\right\}\right)\right)+\\ r_{F_{n}}\left(e_{q_{j}}\right)\cdot s_{D_{m}}\left(e_{q_{j}}\right))\cdot FOG_{n} \end{array}$$

(12) $$CLOUD=\left(r_{E_{n}}\left(\underset{m}{⋃}\left\{d_{1_{m}}\cdots d_{{max}_{m}}\right\}\right)\cdot \psi \left(\underset{n}{⋁}\left\{\underset{m}{⋃}{\left\{d_{1_{m}}\cdots d_{{max}_{m}}\right\}}_{n}\right\}\right)\cdot s_{F_{n}}\left(e_{q_{j}}\right)\right)\cdot CLOUD$$

(13) $$SUB_{j}=\left(r_{B_{q_{j}}}\left(e_{q_{j}}\right)\cdot \phi \left(e_{q_{j}}\right)\cdot s_{OUT_{q_{j}}}\left(e_{q_{j}}\right)\right)\cdot SUB_{j}$$

At that point, all the ACP models may be executed in a concurrent fashion, because of them being all non deterministic, and the encapsulation operator may be applied so as to obtain the sequence of events in a timely manner, as in Equation (14).

In this context, it is to be noted that Equation (14) is similar to Equation (5), therefore, Equation (14) starts with raw data $d_{p_{i}}$ from sensor *p* in the system through channel $IN_{p_{i}}$ towards the publisher *i*. This, in turn, undertakes a unitary processing $\phi (d_{p_{i}})$ and sends them towards edge *m* through channel $A_{i}$. On arrival to the edge, those data are aggregated $\left(d_{1_{m}}\cdots d_{{max}_{m}}\right)$ and if processing is possible by means of edge CNN $\theta (d_{1_{m}}\cdots d_{{max}_{m}})$, then the processed data $e_{q_{j}}$ are forwarded towards the subscriber *j* through channel $B_{j}$, which then carries out a unitary processing $\phi (e_{q_{j}})$ and forwards them out of the system through actuator *q*.

Otherwise, if processing is not possible in edge *m*, then the aggregated data ($d_{1_{m}}\cdots d_{{max}_{m}}$) are forwarded through channel $C_{m}$ to fog *n*, which in turn, gets all aggregated data combined $\left({⋃}_{m}\left\{d_{1_{m}}\cdots d_{{max}_{m}}\right\}\right)$ and tries to process them by means of fog CNN $\chi ({⋃}_{m}\left\{d_{1_{m}}\cdots d_{{max}_{m}}\right\})$. If that is the case, then the process data $e_{q_{j}}$ is sent through channel $D_{m}$ towards edge *m*, which will be led through channel $B_{j}$ to subscriber *j*, and in turn, off the system through channel $OUT_{q_{j}}$ towards actuator *q*.

Furthermore, if processing is not possible in the fog *n*, then it is to be noted that combined data $\left({⋃}_{m}\left\{d_{1_{m}}\cdots d_{{max}_{m}}\right\}\right)$ are sent over to the cloud through channel $E_{n}$, which then gets all combined data $\left({⋁}_{n}\left\{{⋃}_{m}{\left\{d_{1_{m}}\cdots d_{{max}_{m}}\right\}}_{n}\right\}\right)$ and processes them by means of the cloud CNN $\psi ({⋁}_{n}\left\{{⋃}_{m}{\left\{d_{1_{m}}\cdots d_{{max}_{m}}\right\}}_{n}\right\})$, which will result in processed data $e_{q_{j}}$ being forwarded through channel $F_{n}$ towards Fog *n*. This, in turn, will be sent to edge *m* through channel $D_{m}$, which then will be forwarded through channel $B_{j}$ towards subscriber *j*, which in turn, will be forwarded on through channel $OUT_{q_{j}}$ out of the system towards actuator *q*.

(14) $$\begin{array}{c} \sum_{i}\sum_{j}\sum_{p}\sum_{q}\sum_{m}\sum_{n}\partial_{H}\left(PUB_{i}||EDGE_{m}||FOG_{n}||CLOUD||SUB_{j}\right)=\\ (r_{IN_{p_{i}}}\left(d_{p_{i}}\right)\cdot \phi \left(d_{p_{i}}\right)\cdot c_{A_{i}}\left(d_{p_{i}}\right)\cdot \\ (\emptyset ◃\;\theta \left(d_{1_{m}}\cdots d_{{max}_{m}}\right)\;▹c_{C_{m}}\left(d_{1_{m}}\cdots d_{{max}_{m}}\right)\cdot \\ (c_{D_{m}}\left(e_{q_{j}}\right)◃\;\chi \left(\underset{m}{⋃}\left\{d_{1_{m}}\cdots d_{{max}_{m}}\right\}\right)\;▹\\ c_{E_{n}}\left(\underset{m}{⋃}\left\{d_{1_{m}}\cdots d_{{max}_{m}}\right\}\right)\cdot \psi \left(\underset{n}{⋁}\left\{\underset{m}{⋃}{\left\{d_{1_{m}}\cdots d_{{max}_{m}}\right\}}_{n}\right\}\right)\cdot c_{F_{n}}\left(e_{q_{j}}\right)))\\ c_{B_{q_{j}}}\left(e_{q_{j}}\right)\cdot \phi \left(e_{q_{j}}\right)\cdot s_{OUT_{q_{j}}}\left(e_{q_{j}}\right))\cdot \partial_{H}\left(PUB_{i}||EDGE_{m}||FOG_{n}||CLOUD||SUB_{j}\right) \end{array}$$

At that moment, the abstraction operator may be applied so as to attain the external behavior of the model, as in Equation (15). In this context, it is to be said that the abstraction operator masks all internal actions as well as all internal communications.

(15) $$\begin{array}{c} \sum_{i}\sum_{j}\sum_{p}\sum_{q}\sum_{m}\sum_{n}\tau_{I}\left(\partial_{H}\left(PUB_{i}||EDGE_{m}||FOG_{n}||CLOUD||SUB_{j}\right)\right)=\\ r_{IN_{p_{i}}}\left(d_{p_{i}}\right)\cdot s_{OUT_{q_{j}}}\left(e_{q_{j}}\right)\cdot \tau_{I}\left(\partial_{H}\left(PUB_{i}||EDGE_{m}||FOG_{n}||CLOUD||SUB_{j}\right)\right) \end{array}$$

Otherwise, the external behavior of the real system may be denoted by means of ACP, as in Equation (16), where some raw data (*d*) gets into the system from a sensor across an incoming channel ($r_{IN}\left(d\right)$), then gets processed anywhere in the system (that being an edge server, a fog server, or a cloud server) to achieve processed data (*e*), which finally gets out of the system to an actuator across an outgoing channel ($s_{OUT}\left(e\right)$).

(16) $$X=r_{IN}\left(d\right)\cdot s_{OUT}\left(e\right)\cdot X$$

By comparing expressions Equation (15) and Equation (16), it seems to be obvious that they are both recursive expressions multiplied by the same factors. Hence, it may be established that both are rooted branching bisimilar, as they are composed by the same actions and the same branching structure, so Equation (17) applies.

(17) $$\sum_{i}\sum_{j}\sum_{p}\sum_{q}\sum_{m}\sum_{n}\tau_{I}\left(\partial_{H}\left(PUB_{i}||EDGE_{m}||FOG_{n}||CLOUD||SUB_{j}\right)\right)⟷X$$

Therefore, this is a sufficient condition to get a model verified. Hence, the proposed ACP model for fog computing may be considered verified.

## 6. Spin/Promela Scenario

Promela (PROtocol/PROcess MEta LAnguage) is a high level specification language, whose syntax is similar to that of C, aimed at modelling the interactions taking place in distributed systems. It is typically employed as the input language for the Spin (Simple Promela INterpreter) model checker [147]. Promela was designed to deal with non-deterministic processes, communicating through message channels being defined as synchronous or asynchronous. Hence, a Promela model of a concurrent system may be first designed according to certain specifications, and in turn, Spin may be used to verify that such a model produces the desired behavior, in a way that the same input actions in both the real system being modeled and the model itself induce the same output actions.

**Figure 5:**
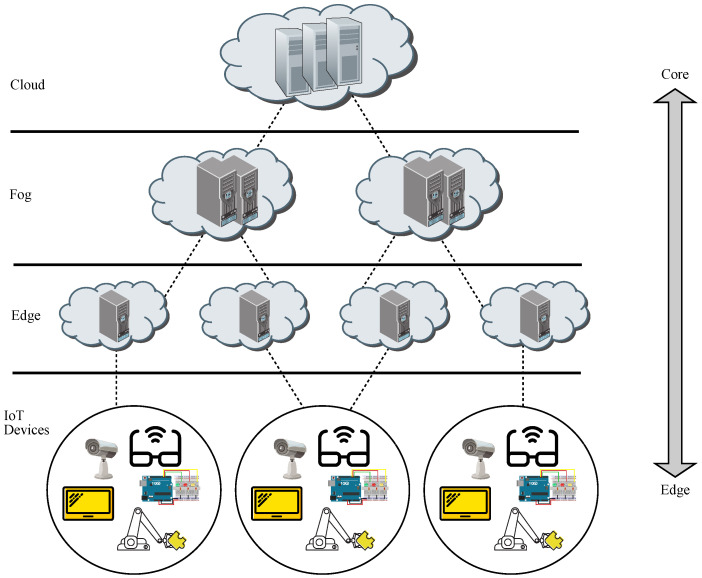
Block diagram for fog computing.

Therefore, a Promela model of a fog computing scenario has been designed in order to further check its behavior with Spin, according to the block diagram shown in Figure 5. There are four different layers exhibited in that picture, such as IoT devices, Edge, Fog, and Cloud, and those are the processes defined in Promela. The expected behavior of the Devices processes are to either initiate or terminate the traffic flow, depending on whether they are connected to the initial sensor or the final actuator. Meanwhile, that of the Edge processes are to either pass a message to a device if they know how to deal with it, or send it to a fog (which is analogous to the Fog processes, as they forward a message to an edge if they know how to handle it, or else send it to the cloud). Additionally, the Cloud process forwards a message to the fog as they are supposed to always know how to manage the message for the higher level of servers.

A Promela model for an edge computing scenario would be similar, as seen in the ACP models, and that is why it is not included herein. As stated above, the edge model would be the same as the fog one, except for taking out the fog layer, thus making direct connections from edge layer to cloud layer.

At this point, the model of a fog computing written in Promela is presented in Algorithm 1, where the type of entities are defined by specifying their communication channels regarding the way they interact in a real fog environment, and then, those types are instantiated accordingly. For recapitulation purposes, they are the four sorts of entities involved, i.e.,

- Devices;
- Edge;
- Fog;
- Cloud.

Regarding the above code, the first two lines are aimed at defining two macros, by means of the keyword #define, to be a constant value so as to make the code clearer to read. Then, the third line defines a message type, by means of the keyword mtype, which abstracts away from the specific values being used, as the corresponding message field is going to be interpreted symbolically, as opposed to numerically, throughout the code. Afterwards, the following six lines declare six global message channels, each being able to store just one message, by means of the keyword chan, in order to transfer data from a source entity to a destination one. Next, there are four declarations of entities, by means of the keyword proctype, all with a parameter value of type byte for identifying each new entity. Each declaration consists of local variables separated by a semicolon and statements, whose causal relationship is indicated by the arrow sign (->) and which may be included into loops or conditional statements. Finally, the entity instantiation is done through the init process, by means of the function run with the identifier.

After running the Promela code in the Spin model checker, different results may be obtained in diverse executions, depending on the seed set to initialize each of them. Hence, every time the program is run, a different outcome is obtained. However, all cases may be grouped into three categories when dealing with arriving messages coming from a sensor, such as that where only the edges handle them all, that where only the edges and the fogs deal with them all, and that where the edges, fogs, and cloud manage them all. It is to be noted that in the first case, the fogs and cloud do not intervene. Meanwhile, in the second case, the cloud does not participate. Likewise, it is to be said that in the second case, the fogs take part at least once, as there may be some transactions where those are not engaged, and similarly, in the third case, the cloud performs at least once, but does not always need to contribute.

For example purposes only, the traces (which actually are displayed as MSC) resulting of three instances obtained by Spin (when running the aforementioned code with diverse seeds) are exposed, where each of them belong to one of the aforementioned categories. It is to be remarked that each traffic flow within the trace is composed by different messages going through diverse neighboring entities, even though all those messages have the same flow identifier, starting with zero, which is the number located right after the string MSG within each label. Furthermore, the name of the channel involved is shown at the beginning of each label, where the source of the traffic flow within each channel is stated by a ! sign and the destination is indicated by a ? sign, both located before the string MSG. Additionally, the horizontal axis depicts a separate entity involved in the trace, whereas the vertical axis exhibits the temporal reference, such that those traces are indeed MSCs, which display changes to each entity on a temporal scale.
**Algorithm 1** Fog model coded in Promela
   #define N 2
   #define INF 99
   mtype = MSG
   chan fromSensor[N*N] = [1] of mtype,byte,byte
   chan toActuator[N*N] = [1] of mtype,byte,byte
   chan Fog2Edge[N*N] = [1] of mtype,byte,byte
   chan Edge2Fog[N*N] = [1] of mtype,byte,byte
   chan Fog2Cloud[N] = [1] of mtype,byte,byte
   chan Cloud2Fog[N] = [1] of mtype,byte,byte
   proctype Devices (byte id) {
      byte x,y,n=0;
      do
      :: n<1 -> fromSensor[id] ! MSG(id,INF); n++
      :: toActuator[id] ? MSG(x,y)
      od
   }
   proctype Edge (byte id) {
      byte x,y;
      do
      :: if
        :: fromSensor[id] ? MSG(x,y) -> if
                                                              :: toActuator[id] ! MSG(x,id)
                                                              :: Edge2Fog[id] ! MSG(x,y)
                                                              fi
        :: Fog2Edge[id] ? MSG(x,y)->toActuator[id] ! MSG(x,y)
        fi
      od
   }
   proctype Fog (byte id) {
      byte x,y;
      do
      :: Edge2Fog[id*2] ? MSG(x,y) -> if
                                                              :: Fog2Edge[id*2+1] ! MSG(x,id*2+1)
                                                              :: Fog2Cloud[id] ! MSG(x,y)
                                                              fi
      :: Edge2Fog[id*2+1] ? MSG(x,y) -> if
                                                                 :: Fog2Edge[id*2] ! MSG(x,id*2)
                                                                 :: Fog2Cloud[id] ! MSG(x,y)
                                                                 fi
      :: Cloud2Fog[id] ? MSG(x,y) -> Fog2Edge[y] ! MSG(x,y)
      od
   }
   proctype Cloud (byte id) {
      byte x,y;
      do
      ::Fog2Cloud[0] ? MSG(x,y) -> select(y:N..N+1) -> Cloud2Fog[1] ! MSG(x,y)
      ::Fog2Cloud[1] ? MSG(x,y) -> select(y:0..1)-> Cloud2Fog[0] ! MSG(x,y)
      od
   }
   init {
      byte i;
      for (i : 0..(N*N-1))
          run Devices (i)
          run Edge(i)
      for (i : 0..(N-1))
          run Fog (i)
      run Cloud(0)
   }


**Figure 6:**
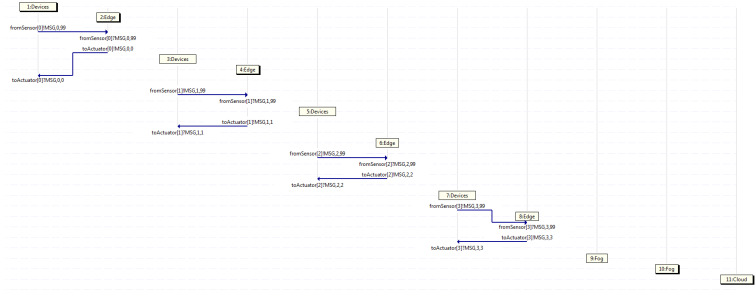
Capture where only edge servers deal with all traffic.

First of all, Figure 6 shows an MSC classified in the first group, where four traffic flows start in different devices, such as 1, 3, 5, and 7, (those being connected to a particular sensor) at diverse time intervals. It may be appreciated that all messages coming from the devices are handled by a certain edge, which in turn, forwards them back to the devices (those being connected to a given actuator). Looking at that MSC, device 1 starts flow 0 sending a message through the channel from the sensor towards edge 2, which handles the message and forwards it back to device 1 through the channel to the actuator. Likewise, the same behavior is shown by the rest of the couples, such as device 3 and edge 4 using flow 1, device 5 and edge 6 taking flow 2, and device 7 and edge 8 employing flow 3.

**Figure 7:**
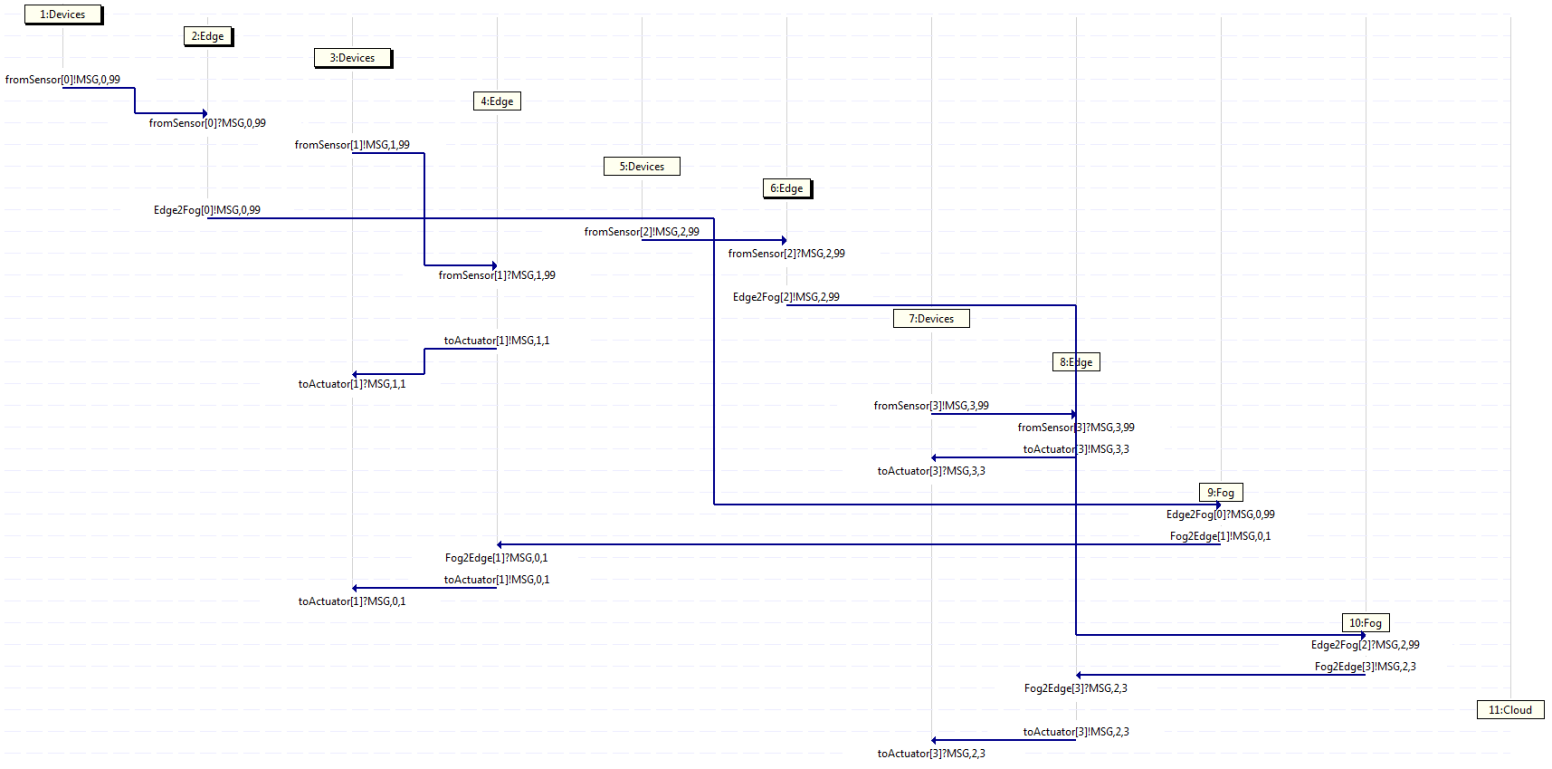
Capture where only edge servers and fog servers deal with all traffic.

After this, Figure 7 depicts an MSC classified in the second group, where four traffic flows start in devices 1, 3, 5, and 7 at diverse time intervals. It may be spotted that a pair of edges handle the messages and send them back to the devices, whereas the other couple of edges do not handle them, but instead, they forward such messages on to a fog, which does handle them. Afterwards, it sends them back to an edge. Watching that MSC, device 1 starts flow 0, forwarding a message through the channel from the sensor towards edge 2, which in turn, forwards it on through channel Edge2Fog towards fog 9, which next, forwards it back to edge 4 through channel Fog2Edge, which then, sends it back to device 3 through the channel to the actuator. Likewise, the same behavior is appreciated by flow 2, which departs from device 5 towards edge 6, and then, towards fog 10, which handles the message and sends it back towards edge 8, and in turn, towards device 7. Otherwise, flow 1 exhibits the behavior described in the first group, as device 3 sends a message to edge 4, which in turn, forwards it back to device 3, whilst so does flow 3, as device 7 forwards a message on to edge 8, which then, sends it back to device 7.

**Figure 8:**
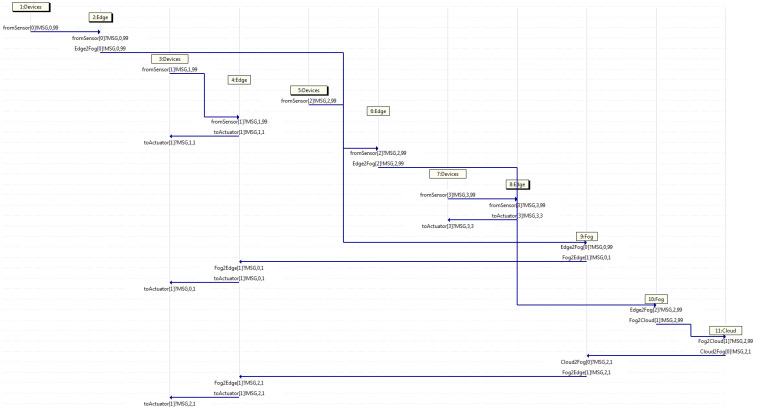
Capture where only edge servers deal with all traffic.

Additionally, Figure 8 displays an MSC classified in the third group, where again, four traffic flows depart in devices 1, 3, 5, and 7 at different time intervals. It may be viewed that a couple of edges handle the messages and forward them back to devices, whilst the other pair of edges are not able to handle them and forward those messages on to fogs. At that point, one fog handle its message and sends it back to an edge, whereas another fog is not able to handle the message, and in turn, that fog forwards the message on to the cloud, which handles the message as it is the higher server in the hierarchy. Studying the MSC, device 5 starts flow 2 sending a message through channel from the sensor towards edge 6, which then, sends it on through channel Edge2Fog towards fog 10, which in turn, forwards it on through channel Fog2Cloud, which handles the message and sends it back through channel Cloud2Fog towards fog 9, which then, forwards it back through channel Fog2Edge towards edge 4, which in turn, sends it back to device 3 through the channel to the actuator. Otherwise, flow 0 exhibits the behavior exposed in the second group, as device 1 forwards a message to edge 2, which then, sends it on to fog 9, which in turn, forwards it back to edge 4, which then, send it back to device 3. Furthermore, flow 1 depicts the behavior explained in the first group, as device 3 sends a message on to edge 4, which in turn, forwards it back to device 3. Meanwhile, flow 3, as in device 7, sends a message on to edge 8, which then, forwards it back to device 7.

## 7. Conclusions

In this paper, we carried out a study modeling a generic edge computing application. First of all, some background was introduced, such as relevant advances in convolutional neural networks, which are key players dealing with edge AI infrastructures and its application to the edge AI concept. Afterwards, background on fog computing has been cited, as well as some of the main trends in the edge development, such the wereable devices, IoT health, industry 4.0 and 5.0, vehicular networks, federated learning, and IoT serverless applications.

Afterwards, modeling was undertaken, starting with an algebraic model by means of a process algebra called ACP of a generic high-level edge computing environment, abstracting away the concepts of sensor, actuator, end device, edge server, and cloud server, taking into account that such a model has been duly specified and verified. This model has later been extended with the addition of fog facilities, which has also been duly specified and verified.

Additionally, a Promela model was undertaken for such a model in order to describe its behavior, which has was later verified with Spin, that being a model checker working with Promela code. Some message sequence charts have also been analyzed, revealing the expected behavior of the model proposed.

In summary, both the algebraic model proposed and verified in ACP and the coding model presented in Promela and verified in Spin meet the requirements related to expected behavior of a generic edge computing environment, and so does its extension to a generic fog computing environment.

## Abbreviations

The following abbreviations are used in this manuscript:

ACP	Algebra of Communicating Processes
AI	Artificial Intelligence
AIoT	Artificial Intelligence of Things
ANN	Artificial Neural Networks
AR	Augmented Reality
Baas	Backend as a Service
CL	Centralized Learning
CNN	Convolutional Neural Networks
CPS	Cyber Physical Systems
DCE	Digital Circular Economy
DL	Deep Learning
DNN	Deep Neural Networks
DNS	Domain Name System
DT	Digital Twins
FaaS	Function as a Service
FDT	Formal Description Techniques
FL	Federated Learning
G-IoT	Green Internet of Things
GRU	Gated Recurrent Units
HFCL	Hybrid Federated Centralized Learning
IIoT	Industrial Internet of Things
IoMT	Internet of Medical Things
IoT	Internet of Things
LAN	Local Area Network
LSTM	Long Short-Term Memory
MEC	Multi-Access Edge Computing
MSC	Message Sequence Chart
MSG	Message
ML	Machine Learning
MR	Mixed Reality
Pub/Sub	Publisher/Subscriber
PS	Parameter Server
PROMELA	PROtocol/PROcess MEta LAnguage
QoS	Quality of Service
RAN	Radio Access Network
RNN	Recurrent Neural Networks
SPIN	Simple Promela INterpreter
V2R	Vehicle to Roadside Unit
V2V	Vehicle to Vehicle
VANET	Vehicular Ad-hoc Network
VEC	Vehicular Edge Computing
VR	Virtual Reality
WAN	Wide Area Network

## References

[1] Carvalho G., Cabral B., Pereira V., Bernardino J. (2021). Edge computing: Current trends, research challenges and future directions. Computing.

[2] Cao K., Liu Y., Meng G., Sun Q. (2020). An Overview on Edge Computing Research. IEEE Access.

[3] A 2021 Perspective on Edge Computing. https://atos.net/wp-content/uploads/2021/08/atos-2021-perspective-on-edge-computing-white-paper.pdf/.

[4] Rahimi H., Picaud Y., Singh K., Madhusudan G., Costanzo S., Boissier O. (2021). Design and Simulation of a Hybrid Architecture for Edge Computing in 5G and Beyond. IEEE Trans. Comput..

[5] Agarwal G.K., Magnusson M., Johanson A. (2020). Edge AI Driven Technology Advancements Paving Way towards New Capabilities. IEEE Int. J. Innov. Technol. Manag..

[6] Xu Z., Liu W., Huang J., Yang C., Lu J., Tan H. (2020). Artificial Intelligence for Securing IoT Services in Edge Computing: A Survey. Secur. Commun. Netw..

[7] Hamdan S., Ayyash M., Almajali S. (2020). Edge-Computing Architectures for Internet of Things Applications: A Survey. Sensors.

[8] Mrabet H., Belgith S., Alhomoud A., Jemai A. (2020). A Survey of IoT Security Based on a Layered Architecture of Sensing and Data Analysis. Sensors.

[9] Fokkink W. (2007). Introduction to Process Algebra.

[10] Ben-Ari M. (2008). Principles of the Spin Model Checker.

[11] Smoliński M. (2017). Resolving Classical Concurrency Problems Using Outlier Detection. J. Appl. Comput. Sci..

[12] Ozkaya M. (2018). Do the informal & formal software modeling notations satisfy practitioners for software architecture modeling?. Inf. Softw. Technol..

[13] Yu Z., Ouyang J., Li S., Peng X. (2017). Formal modeling and control of cyber-physical manufacturing systems. Adv. Mech. Eng..

[14] Hofer-Schmitz K., Stojanovic B. Towards Formal Methods of IoT Application Layer Protocols. Proceedings of the 12th CMI Conference on Cybersecurity and Privacy.

[15] Guizzardi G. (2005). Ontological Foundations for Structural Conceptual Models. Ph.D. Thesis.

[16] Gleirscher M., Marmsoler D. (2020). Formal Methods in Dependable Systems Engineering: A Survey of Professionals from Europe and North America. Empir. Softw. Eng..

[17] Casale G., Gribaudo M., Serazzi G. (2010). Tools for Performance Evaluation of Computer Systems: Historical Evolution and Perspectives. Performance Evaluation of Computer and Communication Systems. Milestones and Future Challenges.

[18] Molero X., Juiz C., Rodeño M. (2004). Evaluación y Modelado del Rendimiento de los Sistemas Informáticos.

[19] Iqbal I.M., Adzkiya D., Mukhlash I. Formal verification of automated teller machine systems using SPIN. Proceedings of the AIP Conference.

[20] Choi R.Y., Coyner A.S., Kalpathy-Cramer J., Chiang M.F., Campbell P. (2020). Introduction to Machine Learning, Neural Networks, and Deep Learning. Transl. Vis. Sci. Technol..

[21] Hart G.L.W., Mueller T., Toher C., Curtarolo S. (2021). Machine learning for alloys. Nature.

[22] Wichert A., Sa-Couto L. (2021). Machine Learning—A Journey to Deep Learning.

[23] Teslyuk V., Kazarian A., Kryvinska N., Tsmots I. (2021). Optimal Artificial Neural Network Type Selection Method for Usage in Smart House Systems. Sensors.

[24] Poggio T., Mhaskar H., Rosasco L., Miranda B., Liao Q. (2019). Why and When Can Deep-but Not Shallow-networks Avoid the Curse of Dimensionality: A Review. Int. J. Autom. Comput..

[25] CNN vs. RNN vs. ANN—Analyzing 3 Types of Neural Networks in Deep Learning. https://www.analyticsvidhya.com/blog/2020/02/cnn-vs-rnn-vs-mlp-analyzing-3-types-of-neural-networks-in-deep-learning/.

[26] Rehmer A., Kroll A. On the vanishing and exploding gradient problem in Gated Recurrent Units. Proceedings of the 21st IFAC World Congress.

[27] Véstias M.P. (2019). A Survey of Convolutional Neural Networks on Edge with Reconfigurable Computing. Algorithms.

[28] Cho K.O., Jang H.J. (2020). Comparison of different input modalities and network structures for deep learning-based seizure detection. Sci. Rep..

[29] Li Y., Xie X., Gool L., Timofte R. (2019). Learning Filter Basis for Convolutional Neural Network Compression. IEEE Int. Conf. Comput. Vis. (ICCV).

[30] Azulay A., Weiss Y. (2019). Why do deep convolutional networks generalize so poorly to small image transformations?. J. Mach. Learn. Res..

[31] Li L., Ma L., Jiao L., Liu F., Sun Q., Zhao J. (2020). Complex Contourlet-CNN for polarimetric SAR image classification. Pattern Recognit..

[32] Image Classification of Rock-Paper-Scissors Pictures Using Convolutional Neural Network (CNN). https://medium.com/mlearning-ai/image-classification-of-rock-paper-scissors-pictures-using-convolutional-neural-network-cnn-c3d2db127cdb/.

[33] Meier D., Wuthrich M.V. (2020). Convolutional Neural Network Case Studies: (1) Anomalies in Mortality Rates (2) Image Recognition. SSRN.

[34] CS231n Convolutional Neural Networks for Visual Recognition. https://cs231n.github.io/convolutional-networks/.

[35] Wang W., Yang Y., Wang X., Wang W.Z., Li J. (2019). Development of convolutional neural network and its application in image classification: A survey. Opt. Eng..

[36] Ma C., Ren Q., Zhao J. (2021). Optical-numerical method based on a convolutional neural network for full-field subpixel displacement measurements. Opt. Express.

[37] Wang Z., Peng J., Song W., Gao X., Zhang Y., Zhang X., Xiao L., Ma L. (2021). A Convolutional Neural Network-Based Classification and Decision-Making Model for Visible Defect Identification of High-Speed Train Images. J. Sens..

[38] Miles C., Bohrdt A., Wu R., Chiu C., Xu M., Ji G., Greiner M., Weinberger K.Q., Demler E., Kim E.A. (2021). Correlator convolutional neural networks as an interpretable architecture for image-like quantum matter data. Nat. Commun..

[39] Höhlein K., Kern M., Hewson T., Westermann R. (2020). A comparative study of convolutional neural network models for wind field downscaling. Meteorol. Appl..

[40] Liu T., Xie X., Zhang Y. (2021). zkCNN: Zero Knowledge Proofs for Convolutional Neural Network Predictions and Accuracy. Cryptol. ePrint Arch..

[41] Pelletier C., Webb G.I., Petitjean F. (2019). Temporal Convolutional Neural Network for the Classification of Satellite Image Time Series. Remote Sens..

[42] Wasay A., Idreos S. More or Less: When and How to Build Convolutional Neural Network Ensembles. Proceedings of the 9th International Conference on Learning Representation (ICLR 2021).

[43] Su R., Liu T., Sun C., Jin Q., Jennane R., Wei L. (2020). Fusing convolutional neural network features with hand-crafted features for osteoporosis diagnoses. Neurocomputing.

[44] Shaban M., Ogur Z., Mahmoud A., Switala A., Shalaby A., Khalifeh H.A., Ghazal M., Fraiwan L., Giridharan G., Sandhu H. (2020). A convolutional neural network for the screening and staging of diabetic retinopathy. PLoS ONE.

[45] Touloupas G., Lauber A., Henneberger J., Beck A., Lucchi A. (2020). A convolutional neural network for classifying cloud particles recorded by imaging probes. Atmos. Meas. Tech..

[46] Dong H., Butler K.T., Matras D., Price S.W.T., Odarchenko Y., Khatry R., Thompson A., Middelkoop V., Jacques S.D.M., Beale A.M. (2021). A deep convolutional neural network for real-time full profile analysis of big powder diffraction data. Comput. Mater..

[47] Satu S., Ahammed K., Abedin M.Z., Rahman A., Islam S.M.S., Azad A.K.M., Alyami S.A., Moni M.A. (2021). Convolutional Neural Network Model to Detect COVID-19 Patients Utilizing Chest X-ray Images. Mach. Learn. Appl..

[48] Bonomi F., Milito R., Natarajan P., Zhu J. (2014). A platform for internet of things and analytics. Big Data and Internet of Things: A Roadmap for Smart Environments.

[49] Saba U.K., Islam S., Ijaz H., Rodrigueds J. (2021). Planning Fog networks for time-critical IoT requests. Comput. Commun..

[50] Sabireen H., Neelanarayanan V. (2021). A Review on Fog Computing: Architecture, Fog with IoT, Algorithms and Research Challenges. ICT Express.

[51] Ma K., Bagula A., Nyirenda C., Ajayi O. (2019). An IoT-Based Fog Computing Model. Sensors.

[52] Donno M., Tange K., Dragoni N. (2019). Foundations and Evolution of Modern Computing Paradigms: Cloud, IoT, Edge, and Fog. IEEE Access.

[53] Pham L.M., Nguyen T.-T., Hoang T.Q. (2021). Towards an Elastic Fog-Computing Framework for IoT Big Data Analytics Applications. Wirel. Commun. Mob. Comput..

[54] Meena V., Gorripatti M., Praba T.S. (2021). Trust Enforced Computational Offloading for Health Care Applications in Fog Computing. Wirel. Pers. Commun..

[55] Al-khafajiy M., Baker T., Al-Libawy H.A., Maamar Z., Aloqaily M., Jararweh Y. (2019). Improving fog computing performance via Fog-2-Fog collaboration. Future Gener. Comput. Syst..

[56] Karakaya A., Akleylek S. (2021). A novel IoT-based health and tactical analysis model with fog computing. PeerJ Comput. Sci..

[57] de Moura-Donassolo B. (2020). IoT Orchestration in the Fog. Ph.D. Thesis.

[58] Kaur J., Agrawal A., Khan R.A. (2020). Security Issues in Fog Environment: A Systematic Literature Review. Int. J. Wirel. Inf. Netw..

[59] Gharbi C., Hsairi L., Zagrouba E. A Secure Integrated Fog Cloud-IoT Architecture based on Multi-Agents System and Blockchain. Proceedings of the 13th International Conference on Agents and Artificial Intelligence (ICAART 2021).

[60] Alzoubi Y.I., Osmanaj V.H., Jaradat A., Al-Ahmad A. (2021). Fog computing security and privacy for the Internet of Thing applications: State-of-the-art. Secur. Priv..

[61] Toor A., Ismal S.U., Sohail N., Akhunzada A., Boudjadar J., Khattak H.A., Din I.U., Rodrigues J. (2019). Energy and performance aware fog computing: A case of DVFS and green renewable energy. Future Gener. Comput. Syst..

[62] Alenizi F., Rana O. (2020). Minimizing Delay and Energy in Online Dynamic Fog Systems. arXiv.

[63] Nayeri Z.M., Ghafarian T., Javadi B. (2021). Application placement in Fog computing with AI approach: Taxonomy and a state of the art survey. J. Netw. Comput. Appl..

[64] Singh J., Singh P., Gill S.S. (2021). Fog computing: A taxonomy, systematic review, current trends and research challenges. J. Parallel Distrib. Comput..

[65] Caminero A.C., Muñoz-Mansilla R. (2021). Quality of Service Provision in Fog Computing: Network-Aware Scheduling of Containers. Sensors.

[66] Ijaz M., Li G., Wang H., El-Sherbeeny A.M., Awelisah Y.M., Lin L., Koubaa A., Noor A. (2020). Fog computing: Intelligent Fog-Enabled Smart Healthcare System for Wearable Physiological Parameter Detection. Electronics.

[67] Tang C., Xia S., Li Q., Chen W., Fang W. (2021). Resource pooling in vehicular fog computing. J. Cloud Comput..

[68] Gaouar N., Lehsaini M. (2021). Toward vehicular cloud/fog communication: A survey on data dissemination in vehicular ad hoc networks using vehicular cloud/fog computing. Int. J. Commun. Syst..

[69] Sengupta J., Ruj S., Bit S.D. (2021). A Secure Fog-Based Architecture for Industrial Internet of Things and Industry 4.0. IEEE Trans. Ind. Inform..

[70] Ungurean I., Gaitán N.C. (2021). Software Architecture of a Fog Computing Node for Industrial Internet of Things. Sensors.

[71] Ogundoyin S.O., Kamil I.A. (2021). A trust management system for fog computing services. Internet Things.

[72] Al-Khafajiy M., Baker T., Asim M., Guo Z., Ranjan R., Longo A., Puthal D., Taylor M.J. (2020). COMITMENT: A Fog Computing Trust Management Approach. J. Parallel Distrib. Comput..

[73] Solomon F.A.M., Sathianesan G.W. (2020). Fog Level Trust for Internet of Things Devices Using Node Feedback Aggregation. J. Comput. Theor. Nanosci..

[74] Patwary A.A., Naha R.K., Garg S., Battula S.K., Patwary A.K., Aghasian E., Amin M.B., Mahanti A., Gong M. (2021). Towards Secure Fog Computing: A Survey on Trust Management, Privacy, Authentication, Threats and Access Control. Electronics.

[75] Manvi S.S., Gowda N.C., Kecskemeti G. (2019). Trust Management in Fog Computing: A Survey. Applying Integration Techniques and Methods in Distributed Systems and Technologies.

[76] Hussain Y., Zhiqiu H., Akbar M.A., Alsanad A., Alsanad A.A., Nawaz A., Khan I.A., Khan Z.U. (2020). Context-Aware Trust and Reputation Model for Fog-Based IoT. IEEE Access.

[77] Hallappanavar V.L., Birje M.N. (2021). A Reliable Trust Computing Mechanism in Fog Computing. Int. J. Cloud Appl. Comput..

[78] Iqbal R., Butt T.A., Afzaal M., Salah K. (2019). Trust management in social Internet of vehicles: Factors, challenges, blockchain, and fog solutions. Int. J. Distrib. Sens. Netw..

[79] Li W., Wu J., Cao J., Chen N., Zhang Q., Buyya R. (2021). Blockchain-based trust management in cloud computing systems: A taxonomy, review and future directions. J. Cloud Comput..

[80] Rasheed A., Chong P.H.J., Ho I.W., Li X.J., Liu W. (2019). An Overview of Mobile Edge Computing: Architecture, Technology and Direction. Trans. Internet Inf. Syst. (KSII).

[81] Cloud Edge Computing: Beyond the Data Center. https://www.openstack.org/use-cases/edge-computing/cloud-edge-computing-beyond-the-data-center/.

[82] What Is Edge Computing? A Practical Overview. https://viso.ai/edge-ai/edge-computing-a-practical-overview/.

[83] El Fog Pasa a un Segundo Plano en la Internet Industrial de las Cosas. https://www.infoplc.net/plus-plus/tecnologia/item/108281-magazine-16-fog-computing-iic/.

[84] Saad A., Faddel S., Mohammed O. (2019). IoT-Based Digital Twin for Energy Cyber-Physical Systems: Design and Implementation. Energies.

[85] Xu Z., Zhang Y., Li H., Yang W., Qi Q. (2020). Dynamic resource provisioning for cyber-physical systems in cloud-fog-edge computing. J. Cloud Comput. Adv. Syst. Appl..

[86] (2020). ETSI GS MEC 003 v2.2.1. Multi-Access Edge Computing (MEC): Framework and Reference Architecture.

[87] Ali B., Gregory M.A., Li S. (2021). Multi-Access Edge Computing Architecture, Data Security and Privacy: A Review. IEEE Access.

[88] (2021). Edge Computing in the Context of Open Manufacturing.

[89] Fondo-Ferreiro P., Estévez-Caldas A., Pérez-Vaz R., Gil-Castiñeira F., González-Castaño F.J., Rodríguez-García S., Sousa-Vázquez X.R., López D., Guerrero C. Seamless Multi-Access Edge Computing Application Handover Experiments. Proceedings of the IEEE 22nd International Conference on High Performance Switching and Routing (HPSR 2021).

[90] Edge Computing Market. https://www.factmr.com/report/4761/edge-computing-market/.

[91] Krishnasamy E., Varrette S., Mucciardi M. (Partnership for Advanced Computing in Europe—Technical Report, EU). Edge Computing: An Overview of Framework and Applications. https://orbilu.uni.lu/handle/10993/46573.

[92] Song Z. (2020). Self-Adaptive Edge Services: Enhancing Reliability, Efficiency, and Adaptiveness under Unreliable, Scarce, and Dissimilar Resources. Ph.D. Thesis.

[93] Edge AI and Cloud AI Use Cases. https://barbaraiot.com/blog/aiot-the-perfect-union-between-the-internet-of-things-and-artificial-intelligence/.

[94] Rong G., Xu Y., Tong X., Fan H. (2021). An edge-cloud collaborative computing platform for building AIoT applications efficiently. J. Cloud Comput..

[95] Sodhro A.H., Pirbhulal S., Alburquerque V.H.C. (2019). Artificial Intelligence-Driven Mechanism for Edge Computing-Based Industrial Applications. IEEE Trans. Ind. Inform..

[96] Deng S., Zhao H., Fang W., Yin J., Dustdar S., Zomaya A.Y. (2020). Edge Intelligence: The Confluence of Edge Computing and Artificial Intelligence. IEEE Internet Things J..

[97] Wang X., Han Y., Leung V.C.M., Niyato D., Yan X., Chen X. (2020). Edge AI (Artificial Intelligence Applications on Edge).

[98] Debouche O., Mahmoudi S., Mahmoudi S.A., Manneback P., Bindelle J., Lebeau F. (2020). Edge Computing and Artificial Intelligence for Real-time Poultry Monitoring. Procedia Comput. Sci..

[99] Vecchio M., Azzoni P., Menychtas A., Maglogiannis I., Felfernig A. (2021). A Fully Open-Source Approach to Intelligent Edge Computing: AGILE’s Lesson. Sensors.

[100] AI-Based Video Analytics for Pandemic Management. https://www.ntu.edu.sg/rose/research-focus/deep-learning-video-analytics/ai-based-video-analytics-for-pandemic-management/.

[101] Al-Habob A.A., Dobre O.A. (2019). Mobile Edge Computing and Artificial Intelligence: A Mutually-Beneficial Relationship. IEEE TCN.

[102] Wang F., Zhang M., Wang X., Ma X., Liu J. (2020). Deep Learning for Edge Computing Applications: A State-of-the-Art Survey. IEEE Access.

[103] Jin X., Li L., Dang F., Chen X., Liu Y. (2021). A survey on edge computing for wearable technology. Digit. Signal Process..

[104] Covi E., Donati E., Heidari H., Kappel D., Liang X., Payvand M., Wang W. (2020). Adaptive Extreme Edge Computing for Wearable Devices. arXiv.

[105] Silva M.C., da Silva J.C.F., Delabrida S., Bianchi A.G.C., Ribeiro S.P., Silva J.S., Oliveira R.A.R. (2021). Wearable Edge AI Applications for Ecological Environments. Sensors.

[106] Greco L., Ritrovato P., Xhafa F. (2019). An edge-stream computing infrastructure for real-time analysis of wearable sensors data. Future Gener. Comput. Syst..

[107] Salkic S., Ustundag B.C., Uzunovic T., Golubovic E. Edge Computing Framework for Wearable Sensor-Based Human Activity Recognition. Proceedings of the International Symposium on Innovative and Interdisciplinary Applications of Advanced Technologies (IAT 2019).

[108] Hartmann M., Hashmi U., Imran A. (2019). Edge computing in smart health care systems: Review, challenges, and research directions. Trans. Emerg. Telecommun. Technol..

[109] Ray P.P., Dash D., De D. (2019). Edge computing for Internet of Things: A survey, e-healthcare case study and future direction. J. Netw. Comput. Appl..

[110] Abdellatif A.A., Mohamed A., Chiasserini C.F., Tlili M., Erbad A. (2020). Edge Computing For Smart Health: Context-aware Approaches, Opportunities, and Challenges. arXiv.

[111] Pazienza A., Mallardi G., Fasciano C., Vitulano F. Artificial Intelligence on Edge Computing: A Healthcare Scenario in Ambient Assisted Living. Proceedings of the Artificial Intelligence for Ambient Assisted Living (AI*AAL.it 2019).

[112] Sun L., Jiang X., Ren H., Guo Y. (2020). Edge-Cloud Computing and Artificial Intelligence in Internet of Medical Things: Architecture, Technology and Application. IEEE Access.

[113] Qiu T., Chi J., Zhou X., Ning Z., Atiquzzaman M., Wu D.O. (2020). Edge Computing in Industrial Internet of Things: Architecture, Advances and Challenges. IEEE Commun. Surv. Tutor..

[114] Craciunescu M., Chenaru O., Dobrescu R., Florea G., Mocanu S. (2020). IIoT Gateway for Edge Computing Applications. Service Oriented, Holonic and Multi-Agent Manufacturing Systems for Industry of the Future.

[115] Basir R., Qaisar S., Ali M., Aldwairi M., Ashraf M.I., Mahmood A., Gidlund M. (2019). Fog Computing Enabling Industrial Internet of Things: State-of-the-Art and Research Challenges. Sensors.

[116] Liao H., Zhou Z., Zhao X., Zhang L., Mumtaz S., Jolfaei A., Ahmed S.H., Bashir A.K. (2020). Learning-Based Context-Aware Resource Allocation for Edge-Computing-Empowered Industrial IoT. IEEE Internet Things J..

[117] Xu X., Zeng Z., Yang S., Shao H. (2020). A Novel Blockchain Framework for Industrial IoT Edge Computing. Sensors.

[118] Koh L., Orzes G., Jia F. (2019). The fourth industrial revolution (Industry 4.0): Technologies disruption on operations and supply chain management. Int. J. Oper. Prod. Manag..

[119] Javaid M., Haleel A. (2020). Critical Components of Industry 5.0 Towards a Successful Adoption in the Field of Manufacturing. J. Ind. Integr. Manag..

[120] Özdemir V., Hekim M. (2019). Birth of Industry 5.0: Making Sense of Big Data with Artificial Intelligence, “The Internet of Things” and Next-Generation Technology Policy. OMICS J. Integr. Biol..

[121] Sun Z., Zhu M., Zhang Z., Chen Z., Shi Q., Shan X., Yeow R.C.H., Lee C. (2021). Artificial Intelligence of Things (AIoT) Enabled Virtual Shop Applications Using Self-Powered Sensor Enhanced Soft Robotic Manipulator. Adv. Sci..

[122] Fraga-Lamas P., Lopes S.I., Fernández-Caramés T.M. (2021). Green IoT and Edge AI as Key Technological Enablers for a Sustainable Digital Transition towards a Smart Circular Economy: An Industry 5.0 Use Case. IEEE Sens..

[123] (2021). Industry 5.0. Towards a Sustainable, Human-Centric and Resilient European Industry.

[124] Xie R., Tang Q., Wang Q., Liu X., Yu F.R., Huang T. (2019). Collaborative Vehicular Edge Computing Networks: Architecture Design and Research Challenges. IEEE Access.

[125] Raza S., Wang S., Ahmed M., Anwar M. (2019). A Survey on Vehicular Edge Computing: Architecture, Applications, Technical Issues, and Future Directions. Wirel. Commun. Mob. Comput..

[126] Liu L., Chen C., Pei Q., Maharjan S., Zhang Y. (2019). Vehicular Edge Computing and Networking: A Survey. arXiv.

[127] Dharminder D., Kumar U., Gupta P. (2021). Edge based authentication protocol for vehicular communications without trusted party communication. J. Syst. Archit..

[128] Raza S., Liu W., Ahmed M., Anwar M.R., Mirza M.A., Sun Q., Wang S. (2020). An efficient task offloading scheme in vehicular edge computing. J. Cloud Comput..

[129] Abdulrahman S., Tout H., Ould-Slimane H., Mourad A., Talhi C., Guizani M. (2020). A Survey on Federated Learning: The Journey From Centralized to Distributed On-Site Learning and Beyond. IEEE Internet Things J..

[130] An introduction to Federated Learning: Challenges and Applications. https://viso.ai/deep-learning/federated-learning/.

[131] Kairouz P., McMahan H.B., Avent B., Bellet A., Bennis M., Bhagoji A.N., Bonawitz K., Charles Z., Cormode G., Cummings R. (2021). Advances and Open Problems in Federated Learning. Found. Trends Mach. Learn..

[132] Zhang W., Cui X., Finkler U., Saon G., Kayi A., Buysktosunoglu A., Kingsbury B., Kung D., Picheny M. A Highly Efficient Distributed Deep Learning System For Automatic Speech Recognition. Proceedings of the Interspeech.

[133] Elbir A.M., Papazafeiropoulos A.K., Chatzinotas S. (2021). Federated Learning for Physical Layer Design. arXiv.

[134] Kjorveziroski V., Filiposka S., Trajkovic V. (2021). IoT Serverless Computing at the Edge: Open Issues and Research Direction. Computers.

[135] Aslanpour M.S., Toosi A.N., Cicconetti C., Javadi B., Sbarski P., Taibi D., Assunção M., Gill S.S., Gaire R., Dustdar S. Serverless Edge Computing: Vision and Challenges. Proceedings of the Australasian Computer Science Week (ASCW 2021).

[136] Zhang M., Krintz C., Wolski R. (2020). Edge-adaptable serverless acceleration for machine learning Internet of Things applications. J. Softw. Pract. Exp..

[137] Benedetti P., Femminella M., Reali G., Steenhaul K. (2021). Experimental Analysis of the Application of Serverless Computing to IoT Platforms. Sensors.

[138] Wang B., Ali-Eldin A., Shenoy P. (2021). LaSS: Running Latency Sensitive Serverless Computations at the Edge. arXiv.

[139] Ghaemi S., Rouhani S., Belchior R., Cruz R.S., Khazaei H., Musilek P. (2021). A Pub-Sub Architecture to Promote Blockchain Interoperability. arXiv.

[140] Edge Computing and Thermal Management. https://www.qats.com/cms/2020/01/14/edge-computing-and-thermal-management/.

[141] Alcaraz S., Roig P.J., Gilly K., Filiposka S., Aknin N. Formal Algebraic Description of a Fog/IoT Computing Environment. Proceedings of the 24th International Conference Electronics.

[142] Bergstra J.A., Middleburg C.A. (2020). Using Hoare Logic in a Process Algebra Setting. arXiv.

[143] Fokkink W. (2017). Modelling Distributed Systems.

[144] Roig P.J., Alcaraz S., Gilly K., Juiz C., Aknin N. MQTT Algebraic Formal Modelling Using ACP. Proceedings of the 24th International Conference Electronics.

[145] Krishnan R., Lalithambika V.R. (2020). Modeling and Validating Launch Vehicle Onboard Software Using the SPIN Model Checker. J. Aerosp. Inf. Syst..

[146] Ponomarenko A.A., Garanina N.O., Staroletov S.M., Zyubin V.E. Towards the Translation of Reflex Programs to Promela: Model Checking Wheelchair Lift Software. Proceedings of the IEEE 22nd International Conference of Young Professionals in Electron Devices and Materials (EDM).

[147] Comini M., Gallardo M.M., Villanueva A. (2021). A denotational semantics for PROMELA addressing arbitrary jumps. arXiv.

